# The utilisation of emergency point-of-care ultrasound in a tertiary hospital emergency department in East London, South Africa

**DOI:** 10.1016/j.afjem.2024.05.002

**Published:** 2024-06-04

**Authors:** Oscar Biggs, Luan Taljaard, Daniël Jacobus Van Hoving, Meeren Rugunanan

**Affiliations:** a Frere Hospital Department of Emergency Medicine, East London, South Africa; b Division of Emergency Medicine, Stellenbosch University, Cape Town, South Africa

**Keywords:** Emergency medicine, Emergency department, Emergency point-of-care ultrasound, Eastern Cape, South Africa

## Abstract

**Introduction:**

Emergency departments are the primary entry point for emergencies in the public healthcare system. Resource constraints burden a large proportion of the public hospital emergency departments, which includes limited access to radiological services. Emergency point-of-care ultrasound provides a tool capable of bridging this gap. The Eastern Cape is yet to describe the utilisation of emergency point-of-care ultrasound in any of its emergency departments.

**Methods:**

Frere Hospital initiated a clinical audit to assess the utilisation of emergency point-of-care ultrasound in its emergency department in 2022. This study was a retrospective review of the audit between 01 November 2022 until 28 February 2023. Data from the handwritten register regarding patient's presenting complaints and provisional diagnoses was also captured during the study period to draw comparisons between burden of disease and use of emergency point-of-care ultrasound.

**Results:**

A total of 9501 patients attended Frere Hospital's emergency department over the study period with 492 emergency point-of-care ultrasounds performed (overall utilisation rate 5.2 %). The five credentialed emergency point-of-care ultrasound providers performed the majority (*n* = 360, 73.2 %) of the applications, compared to 132 (26.8 %) performed by the seven non-credentialed providers. The extended focused abdominal sonography in trauma (eFAST) was the most frequently performed application (*n* = 140, 28.5 %).

**Conclusion:**

Emergency point-of-care ultrasound is underutilised in Frere Hospital's emergency department. The varied casemix requires upskilling of clinicians in emergency point-of-care ultrasound to suit the burden of disease experienced in the department. Ongoing emergency point-of-care ultrasound training, credentialing and research is important to ensure appropriate and quality emergency point-of-care ultrasound utilisation.

## African relevance

• Quality emergency health care is not a reality in many low- and middle-income countries.
• The WHO (World Health Organisation) estimates 60 % of the world's population does not have access to basic radiological services.
• Emergency point-of-care ultrasound is growing in its utilisation in Africa.
• Describing its utilisation could guide future ePoCUS training and development in the Eastern Cape and other parts of Southern Africa.

## Introduction

South Africa is a middle-income country and experiences a quadruple burden of disease that includes maternal, newborn and child health, non-communicable diseases, HIV/AIDS and tuberculosis, violence and trauma-related injuries [1]. Emergency Departments (EDs) are the primary entry point for emergencies in the public healthcare system [1]. Limited radiological services are one of the challenges faced by resource constrained public hospital EDs [2].

The World Health Organisation (WHO) estimates that 60 % of the world's population does not have access to basic radiological investigations such as X-rays [3]. Emergency point-of-care ultrasound (ePoCUS) presents a diagnostic tool capable of filling this gap; with its utilisation in developing countries being described as essential by the WHO [2,4,5].

The International Federation of Emergency Medicine (IFEM) defines ePoCUS as a “diagnostic or procedural guidance ultrasound that is performed by a clinician during a patient encounter to help guide the evaluation and management of the patient” [6]. Ultrasound is portable, non-ionizing, non-invasive, repeatable, and cost-effective making ePoCUS an ideal tool in the ED [2,4]. ePoCUS has excellent skill acquisition and retention by all levels of healthcare providers after minimal training [5,7,8]. Since the inception of formal emergency medicine training in South Africa in 2003, ePoCUS has become an asset in many trained emergency physicians' hands.

The Emergency Medicine Society of South Africa (EMSSA) is one organization that provides ePoCUS training in South Africa and the only organization to offer credentialing for ePoCUS [7,9]. In 2021, EMSSA revised its guidelines for the training and credentialing of ePoCUS providers, which included a new course format and course content [9]. The core curriculum consists of 6 applications: image acquisition and optimisation, extended focused assessment with sonography in trauma (eFAST), focused abdominal aorta ultrasound, basic cardiac ultrasound (including limited compression ultrasound to rule in deep venous thrombosis in patients with a suspicion of a pulmonary embolism), basic lung ultrasound and ultrasound-guided vascular access.

The majority of the EMSSA core ePoCUS curriculum matches the local burden of disease, with trauma (eFAST), acute respiratory complaints and chest pain (focused lung/echocardiography ultrasound) being leading presentations to South African EDs [[10], [11], [12]]. Importantly, ePoCUS curriculum is not solely driven by burden of disease. Multiple factors constitute curriculum development such as time required to teach, ease of application use, ability to reproduce accurate findings and impact on patient management. The current EMSSA ePoCUS curriculum aims to give clinicians a framework to develop their skills in ePoCUS [9].

The utilisation of ePoCUS in South Africa is poorly described. A 2023 study described the use of ePoCUS in Tshwane, South Africa; revealing that 88 % of the 117 participants used ePoCUS in their practice. The majority (58.1 %) of these providers considered themselves general practitioners working in EDs and concerningly, only 36.8 % of the participants had attended the EMSSA core ePoCUS course [14]. ePoCUS is anecdotally utilised by both credentialed and non-credentialed clinicians, often beyond the scope of their training [15].

The Eastern Cape is yet to describe the utilisation of ePoCUS in any of its EDs. The study objectives were i) to describe the utilisation of ePoCUS in a non-emergency physician run tertiary hospital ED in the Eastern Cape province of South Africa, and ii) to compare the casemix of patients with the utilisation of ePoCUS.

## Methods

We present the findings of a clinical audit performed at Frere Hospital over a 4-month period (01 November 2022 - 28 February 2023).

Frere Hospital ED initiated an audit to assess the utilisation of ePoCUS within the ED in 2022 as a quality improvement initiative to determine current utilisation and to identify areas of potential underutilisation which could guide future ePoCUS training.

Frere Hospital is a tertiary hospital in East London, South Africa that serves a population of over 890,000 people [16]. The department serves East London, as well as surrounding rural areas up to 50 km away. The ED is staffed by twelve Medical Officers (MOs) which includes the acting Head of Department (HoD) and does not have an ED specialist. All MOs, including the HoD, were full-time employees in the ED and worked an equal amount of daytime and commuted overtime hours during the audit period. The HoD was responsible for academic teaching which included ePoCUS training/oversight. The department has access to 24-hour radiological services in the form of X-rays and CT scanning. Two ultrasound machines are permanently located in the ED. Both machines worked appropriately during the audit period. Each machine had a curvilinear, linear, and phased array transducer. No MO at the time of the audit had access to or used a handheld ultrasound device.

ED MOs were requested to complete an ePoCUS audit form (supplementary material) for all patients on whom they performed an ePoCUS application(s) at the time of consultation. MOs were encouraged to complete a form for each specific ePoCUS application performed on a single patient, thus allowing more than one application to be performed per patient. No additional forms were completed for repeat ePoCUS applications performed on the same patient for progress/monitoring purposes to prevent duplicate entries. Completed audit forms were placed in the patient's folder and a carbon copy was stored in a sealed box in the department. The HoD collected forms weekly and stored them in the department office for review. At the beginning of the audit, five of the twelve MOs had passed the EMSSA core ePoCUS credentialing examination with one MO becoming credentialed during the audit period. One MO was an examiner and three were instructors (EMSSA ePoCUS accredited) with two having completed the advanced EMSSA ePoCUS course. The advanced course includes three mandatory modules (focused cardiac ultrasound with hemodynamic assessment, deep vein thrombosis extended compression ultrasound and advanced thoracic/lung ultrasound) with a choice of two additional modules (regional blocks/ hepatobiliary and genito-urinary tract/gastro-intestinal/focused obstetrics and gynaecology/transcranial doppler and ocular ultrasound) [9]. The remaining MOs completed the EMSSA core ePoCUS course. The MOs were predominantly classified as grade 1 (< five years as an independent practitioner) with one being a grade 2 (5–10 years as an independent practitioner) and one MO a grade 3 (> 10 years as an independent practitioner). The 12 MOs consult all patients presenting to the ED, either directly or by overseeing an intern. Audit forms that weren't signed and forms with more than 50 % missing data points were excluded.

The handwritten patient registers within the ED were reviewed to determine the casemix of patients presenting to the ED. The handwritten register is completed by a nurse who enters the data including time and date of presentation, presenting complaints, provisional diagnoses and disposition . Once all the data were collected from the handwritten registers, ICD-10 (International Classification of Diseases 10th Revision) codes were assigned by the researchers based on the provisional diagnosis from the register. An experienced and credentialed ePoCUS provider in our setting (> 2 years experience) then assigned potential ePoCUS applications to the collated casemix. The single most appropriate ePoCUS application for each case was selected. The reason for only a single application being assigned was to reduce potential over-estimation in the number of potential applications allocated. The criteria used to assign potential applications was based on the presenting complaints and provisional diagnosis gained from the handwritten register. This was independently repeated by a second researcher for 10 % (*n* = 950) of cases to increase the validity of results. This is a well-known and accepted method supported by Cochrane. The second researcher is a credentialed ePoCUS provider and instructor for EMSSA, they randomly selected 10 % of the cases from the Excel spreadsheet. Both researchers were blinded from each other's allocations. Discrepancies were noted in 18 cases (1.9 %) with all differences resolved through discussion. If it was found during the review that there were incomplete records pertaining to both the patient's presenting complaint and provisional diagnosis (i.e. if both were missing), no potential ePoCUS application was assigned to the patient. Children (<13 years of age) were excluded from the comparison between the ED casemix and potential ePoCUS applications as children made up a minority of ePoCUS performed with only 2 (0.4 %) ePoCUS applications being recorded.

Data were manually entered into Microsoft Excel and analysed using SPSS Statistics for Windows, Version 28.0 (IBM Corp. Released 2021. Armonk, NY: IBM Corp.). Descriptive statistics are used to describe all variables. Categorical variables are presented as percentages or frequencies while the mean and standard deviation (SD) or median and interquartile range were used for continuous variables.

Ethics approval was granted by the Frere and Cecilia Makiwane Hospitals Research Ethics Committee (Reference number: FCMHREC/A0134/2022).

## Results

A total of 9501 patients attended Frere Hospital's ED over the study period, of which 1131 (11.9 %) were children (<13 years) and 4756 (50.1 %) patients were trauma related. The mean (SD) age of patients was 35.1 (19.1) years and 5240 (55.2 %) were male. Most patients presented between 08h00–15h59 (*n* = 3937, 41.4 %) and on Sundays (*n* = 1791, 18.9 %) (Table 1).

**Table 1:** Demographic data of patients attending Frere Hospital emergency department combined with ePoCUS demographics from November 2022-February 2023.

		Total patients		ePoCUS audit	
		n	%	n	%
**Age (years)**	<13	1131	11.9	2	0.4
	13–18	356	3.8	6	1.2
	>18–30	2258	23.8	124	25.2
	>30–50	**3545**	**37.3**	**186**	**37.8**
	>50	2034	21.4	155	31.5
	Unknown	177	1.9	19	3.9
**Gender**	Male	**5240**	**55.2**	**258**	**52.4**
	Female	4146	43.6	227	46.1
	Unknown	115	1.2	7	1.4
**Presentation**	Trauma	**4756**	**50.1**	129	26.2
	Non-trauma	4745	49.9	**363**	**73.8**
	Unknown	0	0.0	0	0.0
**Day of the week**	Monday	1470	15.5	59	12.0
	Tuesday	1220	12.8	71	14.4
	Wednesday	1154	12.1	64	13.0
	Thursday	1041	11.0	**93**	**18.9**
	Friday	1241	13.1	63	12.8
	Saturday	1583	16.7	76	15.4
	Sunday	**1791**	**18.9**	65	13.2
	Unknown	1	0.0	1	0.2
**Time of day**	08h00–15h59	**3937**	**41.4**	161	32.7
	16h00–23h59	3770	39.7	**209**	**42.5**
	00h00–07h59	1721	18.1	121	24.6
	Unknown	73	0.8	1	0.2

Bold font- Highest numbers.

A total of 492 ePoCUS audit forms were completed on 456 patients. All forms were included. The ePoCUS utilisation rate was 5.2 %. ePoCUS applications were performed on 258 (52.4 %) males, while the mean (SD) age of patients was 42.8 (17.6) years. Most of the ePoCUS applications (*n* = 209, 42.5 %; missing *n* = 1) were performed during the first 8 h of the night shift (16h00–23h59) and most were performed on Thursday (*n* = 93, 18.9 %) (Table 1).

The ED experiences a casemix with varying presentations. Trauma and non-trauma caseloads were similar during the audit seeing 4756 patients (50.1 %) and 4745 (49.9 %) respectively. Presenting complaints and provisional diagnosis as recorded by nursing staff are reflected below (Table 2).

**Table 2:** The top ten provisional diagnosis (according to International Classification of Diseases 10th Revision (ICD-10) codes) and presenting complaints of patients presenting to the emergency department of Frere Hospital over a 4-month period.

Trauma (4756)					
Adult (4193)^a^					
Presenting complaint	n	%	Provisional Diagnosis	n	%
Lower limb pain after fall	412	10.0	Soft tissue disorder	1710	41.1
Laceration on head due to assault	408	9.9	Unspecified open wound	1049	25.2
Penetrating trauma to limb(s)	386	9.3	Fracture (unspecified)	630	15.2
MVA - isolated injury	384	9.3	Rhabdomyolysis (Crush injury)	75	1.8
Penetrating trauma to chest/abdomen	311	7.5	Dislocation(unspecified)	70	1.7
Blunt trauma to limb(s)	299	7.2	Unspecified pneumothorax	66	1.6
Blunt trauma to head	284	6.9	Head injury (unspecified)	61	1.5
Fall on an outstretched hand	201	4.9	Foreign body (material unspecified)	52	1.3
PVA - isolated injury	198	4.8	Open fracture(position unspecified)	51	1.2
Community assault	123	3.0	Unspecified eye injury of unspecified eye and orbit	39	0.9
Other	1125	27.2	Other	355	8.5
Unknown	62	1.5	Unknown	35	0.8


Paediatrics (471)					
Presenting complaint	n	%	Provisional Diagnosis	n	%
Fall on an outstretched hand	92	19.7	Soft tissue disorder	229	49.2
Lower limb pain after fall	51	10.9	Fracture (unspecified)	97	20.9
Burns	38	8.2	Unspecified open wound	62	13.3
PVA - isolated injury	30	6.4	Foreign body (material unspecified)	26	5.6
Fall from a height	27	5.8	Head injury(unspecified)	9	1.9
Penetrating trauma to limb(s)	26	5.6	Dental trauma(unspecified)	6	1.3
Blunt trauma to head	23	4.9	Unspecified eye injury of unspecified eye and orbit	5	1.1
Laceration after fall	21	4.5	Unspecified effects of drowning and nonfatal submersion, initial encounter	4	0.9
Dog bite	20	4.3	Cellulitis(unspecified)	3	0.6
MVA - isolated injury	19	4.1	Skull fracture (open/depressed)	3	0.6
Other	119	25.5	Other	21	4.5
Unknown	5	1.1	Unknown	6	1.3


Non - trauma (4745)^b^					
Adult (4000)					
Presenting complaint	n	%	Provisional Diagnosis	n	%
Dyspnoea	536	13.4	Miscarriage (spontaneous)	190	6.0
Undifferentiated abdominal pain	420	10.5	Lower respiratory tract infection (unspecified)	183	5.8
Chest pain	223	5.6	Psychosis	172	5.4
Seizures	209	5.2	Infectious Gastroenteritis (unspecified)	168	5.3
Abnormal uterine bleeding	196	4.9	Gastritis	161	5.1
Abnormal behaviour	180	4.5	Urinary tract infection(site not specified)	141	4.5
Vomiting	158	4.0	Muscle spasm	121	3.8
Headache	148	3.7	Stroke(unspecified)	99	3.1
Generalised body weakness	136	3.4	Suicide attempt	97	3.1
Epigastric pain	113	2.8	Cancer (local/metastatic)	92	2.9
Other	1672	41.9	Other	1735	54.9
Unknown	9	0.2	Unknown	841	21.0


Paediatrics (660)					
Presenting complaint	n	%	Provisional Diagnosis	n	%
Dyspnoea	105	15.9	Infectious Gastroenteritis (unspecified)	141	24.8
Vomiting	104	15.8	Lower respiratory tract infection (unspecified)	71	12.5
Pyrexia	68	10.3	Acute upper respiratory tract infection	44	7.7
Seizures	63	9.6	Febrile seizures (unspecified)	24	4.2
Abdominal pain	41	6.2	Unspecified asthma exacerbation	23	4.0
Coughing	40	6.1	Acute bronchiolitis unspecified	23	4.0
Diarrhoea	33	5.0	Tonsilitis unspecified	20	3.5
Ingestion	20	3.0	Poisoning by unspecified drugs, medicaments, and biological substances(accidental)	17	3.0
Rash	18	2.7	Otitis media(unspecified ear)	17	3.0
Soft tissue swelling	16	2.4	Epilepsy, intractable, with status epilepticus	16	2.8
Other	151	22.9	Other	172	30.3
Unknown	1	0.2	Unknown	92	13.9

a Missing age 92.

b Missing age 85/ Other- different presenting complaint or provisional diagnosis from those mentioned in top 10 / Unknown-missing information from register and unable to record provisional diagnosis or presenting complaint.

Five credentialed ePoCUS providers performed the majority (*n* =360, 73.2 %) of the applications, compared to 132 (26.8 %) performed by the seven non-credentialed ePoCUS providers (supplementary material). eFAST was the most frequently performed application (*n* =140, 28.5 %), followed by focused echocardiography (*n* =115, 23.4 %), and focused obstetrics and gynaecology applications (*n* = 62,12.6 %). Abnormal findings were recorded in 340 (69.1 %) applications (Table 3).

**Table 3:** Number and percentage of performed ePoCUS applications and recorded abnormal findings per application.

ePoCUS applications	ePoCUS applications performed		Abnormal findings	
	n	%	n	%
Extended Focused Assessment with Sonography in Trauma (eFAST)	140	28.5	65	46.4
Focused echocardiography	115	23.4	87	75.7
Focused obstetrics and gynaecology scan^a^	62	12.6	57	91.9
Focused Hepatobiliary scan	57	11.6	40	70.2
Focused Lung scan	35	7.1	32	91.4
Limited compression ultrasound of lower limbs (LCUS)	28	5.7	14	50.0
Focused assessment with sonography for HIV associated TB (FASH)	17	3.5	12	70.6
Procedure guidance (CVC/IV/Paracentesis)^b^	16	3.3	16	-
Rapid ultrasonography for shock and hypotension (RUSH)^c^	6	1.2	6	100.0
Kidney/ureter/bladder ultrasound (KUB)	5	0.8	4	80.0
Focused testes ultrasound	4	0.8	4	100.0
Focused assessment of abdominal aorta	4	0.8	1	25.0
Focused ocular assessment	1	0.2	0	0.0
Focused thyroid ultrasound	1	0.2	1	100.0
Focused musculoskeletal ultrasound	1	0.2	1	100.0

ePoCUS: emergency point-of-care ultrasound.

a ePoCUS for the female patient to assist in the assessment of abdominal pain, vaginal bleeding, shock, pregnancy and pre-term labour” .

b CVC- Central venous catheterisation/ IV- Intravenous catheterisation.

c RUSH protocol includes scans for heart contractility, IVC, abdominal FAST views, aorta assessment and lungs views for pneumothorax in a shocked patient.

**Fig. 1:**
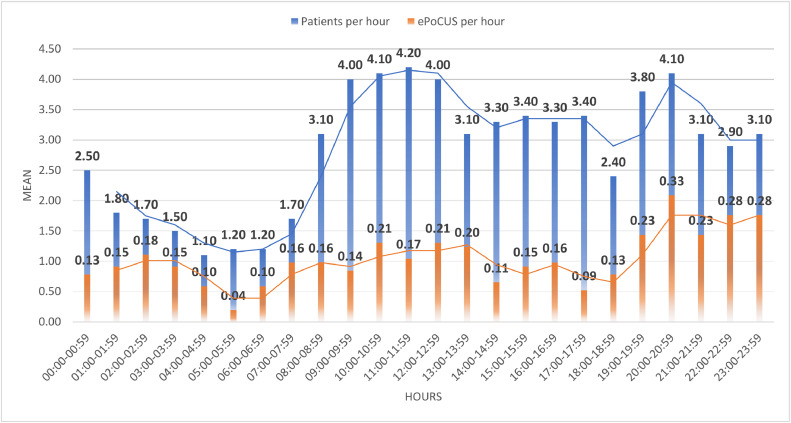
Emergency department patient load per hour with moving mean and emergency point-of-care ultrasound per hour with moving mean.

The department saw a mean of 79 patients per day (mean 3.3 patients per hour). The distribution of patients followed a bimodal pattern, with a peak between 09h00–13h00 (mean 4.1 patients per hour) and a second peak from 19h00–21h00 (mean 3.95 patients per hour) (Fig. 1). A mean of 4 ePoCUS applications were documented per day (mean of 0.17 per hour) which peaked between 10h00–14h00 (mean 0.20 per hour) and from 19h00–00h00 (mean 0.27 per hour) (Fig. 1).

Regarding the top 5 applications (Table 4), a total of 409 (27.1 %) were performed in comparison to the 1508 potential applications (no missing data/exclusions during allocation on potential applications). The closest relationship between actual (*n* = 103) and potential applications (*n* = 326) was during the 00h00–07h59 period with 31.6 % of applications performed. A total of 804 (53.3 %) of the potential applications would have been in males and most would have been in the 30–50 age group (*n* = 536, 35.5 %).

**Table 4:** Top five Emergency point-of-care ultrasound (ePoCUS) applications performed with comparison of potential applications that may have been performed according to the casemix review.

Type of scan	(n) performed	(n) potential applications	(%) actual vs potential applications
Extended focused assessment with sonography in trauma(eFAST)	140	529	26.5
Focused Echocardiography	115	168	68.5
Focused lung ultrasound	35	452	7.7
Focused obstetrics and gynaecology scan	62	244	25.4
Focused hepatobiliary scan	57	115	49.6

## Discussion

The utilisation of ePoCUS applications resembled the casemix in the ED of Frere Hospital. The most frequently used ePoCUS were the eFAST, focused echocardiography, and focused obstetrics and gynaecology applications. A discrepancy was noticed between actual ePoCUS applications performed and assigned potential applications. Almost three-quarters (73 %) of the ePoCUS applications were performed by credentialed ePoCUS providers. ePoCUS were performed in 5.2 % of ED patients. Limited data exists with regards to ePoCUS utilisation rate as compared to ED caseload. An Australian study reported that 2.1 % of ED patients received an ePoCUS [17]. A 2017 multicentre French study that described the prevalence and use of ePoCUS in 50 EDs indicated an ePoCUS utilisation rate of 5.0 % [18].

The hourly distribution of ePoCUS applications performed resembled the hourly patient load in the department. Previous ePoCUS research performed in South Africa showed the majority of ePoCUS applications were performed during office hours, with the researchers suggesting a correlation to specialist emergency physician oversight during these hours [19]. Our data showed a bimodal pattern of distribution, with peaks related to patient load and staff handover rounds. A possible explanation for this is that doctors gradually see less patients per hour during a shift. Additionally, a spike in clinician-patient consults per hour is typically highest in the first few hours of their shift [20].

The highest proportion of actual to potential applications performed (31.6 %) were during the 00h00–07h59 period, which correlates to the quietest time in the ED. The majority (19.0 %) of applications were performed on a Thursday, which was the quietest day. It is possible that reduced patient load resulted in more frequent ePoCUS utilisation as it allowed each MO more time per consultation. The contrary could also be argued, where appropriate and skilled ePoCUS utilisation could aid the clinician in timely diagnostic decision making and therefore add value during busy periods in the ED.

The most frequently performed application was the eFAST (28.5 %), which correlates with local and international research [17,19,21]. The reason for this could be the high trauma burden experienced, as well as the fact that the eFAST application has been in use since the late 1990′s and historically is the most well-known ePoCUS application across both emergency medicine and surgical disciplines [22]. Focused echocardiography was the second most utilised ePoCUS application (23.4 %). This is probably a result of the high prevalence of undifferentiated acute dyspnoea presentations (*n* = 536, 13.4 %). This is less than the 40 % of focused emergency echocardiography in resuscitation (FEER) described over 6 months in another South African province, but similar to a prospective Australian study where 22 % of ePoCUS utilised were focused echocardiography [17,19]. A recently published 2023 study on perceived ePoCUS utilisation in Gauteng province, the eFAST was the second most commonly used application (79.5 %) followed by focused echocardiography (73.5 %). Interestingly, the most stated indication for the use of ePoCUS was to confirm the presence of intra-uterine pregnancies (80.3 %) [14].

Concerningly, a significant percentage of adult patients who presented with an acute respiratory illness did not receive a lung ePoCUS, with only 7.7 % of potential ultrasounds being performed. It is noted that there is a discrepancy in the number of focused echocardiography (*n* = 115) and lung ultrasound (*n* = 35) applications which could be seen as “hand-in-hand” applications in many patients presenting with acute undifferentiated dyspnoea. This is in keeping with a 2022 multicentre study done in Australia where lung ultrasound accounted for only 10 % of cases [23]. A survey in 2022 looked at perceived use of ePoCUS in Africa and found that 40.6 % of respondents used lung ultrasound in their daily practice [15]. We believe there are multiple reasons for this potential underutilisation in this audit. The studied ED has 24-hour access to radiological facilities (X-ray/CT scan) and it is possible that many physicians find a chest radiograph more helpful in evaluating lung pathology due to lack of appropriate knowledge and training in lung ultrasound. Focused lung ePoCUS is a growing application and the benefits, especially in the critically ill patient with pneumothorax, consolidation, diffuse interstitial syndrome and pleural effusion, are well documented and proven superior to plain radiographs [24]. Lung ePoCUS also has the benefit of being non-ionising and rapidly applied at the bedside.

During the audit only 2 (0.4 %) ePoCUS applications were performed on children. This is an interesting finding given the high caseload of paediatric respiratory cases . Many of these patients would have been likely to receive a chest x-ray as part of their diagnostic workup. A systematic review done on lung ultrasounds has showed no statistical difference between chest x-ray and lung ultrasound in diagnosing pneumonia [25]. The low utilisation of ePoCUS in children may highlight a lack of training and knowledge in the evolving field of paediatric ePoCUS.

The hepatobiliary and the focused obstetrics and gynaecology application were frequently performed (11.6 % and 12.6 %). This is a reflection of the casemix, where undifferentiated abdominal pain (*n* = 420, 10.5 %) and abnormal uterine bleeding (*n* = 196, 4.9 %) were common complaints.

FASH applications were only utilised in 3.5 % of all performed ePoCUS. This is surprising given the high numbers of TB and HIV in this specific ED and in the rest of South Africa [1,26]. This is in contrast with a study from the Western Cape, where 43.2 % of respondents suggested FASH applications would match their perceived burden of disease [13].

Ultrasound guided central venous catheterisation (CVC) is considered standard of care. Only 16 (3.3 %) applications recorded were for procedure guidance, of which 6 out of 492(1.2 %)) were used for CVC. Reasons behind this low utilisation rate need to be explored and highlights an area for training.

An increased ePoCUS utilisation was observed among MOs who were credentialed. These credentialed clinicians may be more skilled in ePoCUS and therefore experience greater benefit in incorporating it in their clinical practice, resulting in more frequent utilisation. Credentialing has been controversial at times. Motivation for becoming a credentialed ePoCUS provider may often not relate to skill, but the need to pass the ePoCUS credentialing examination to further one's career in Emergency Medicine. Many experienced ePoCUS providers thus remain uncredentialed but utilise ePoCUS in their practice [27].

The audit aided in suggesting areas of potential ePoCUS underutilisation within Frere hospital ED. A separate qualitative study on ePoCUS guided CVC utilisation must be considered in the future to understand the reasons behind the lower-than-expected utilisation. The specific disease burden necessitates further training and upskilling in applications such as focused lung, focused hepatobiliary and focused obstetrics/gynaecology. This has been achieved through departmental training sessions and encouragement to expand ePoCUS skills by attendance of advanced courses.

## Limitations

The study was an analysis of audit forms completed over 4 months in a single ED. This limits external validity as ePoCUS utilisation may look different in settings with contrasting disease burden and ePoCUS skill level. Data collection was reliant on the manual completion of audit forms and handwritten registers. This had the potential to introduce bias since both are prone to non-completion or missing data, resulting in a sample that might not adequately represent the population. The process of allocating potential ePoCUS applications retrospectively together with a substantial amount of forms being completed by a single person could have introduced confirmation bias. The potential applications may have been underestimated as only one application was allocated per patient while many disease processes require more than one application to give adequate information; e.g., in a patient presenting with undifferentiated shock.

## Conclusion

From the audit there appears to be an overall underutilisation of ePoCUS in the ED of Frere Hospital. The casemix requires upskilling of clinicians in ePoCUS applications suited to the specific disease burden in the department. Ongoing ePoCUS training, credentialing and research is important to ensure appropriate and quality ePoCUS utilisation.

## Dissemination of results

Results have been distributed to relevant Emergency departments and hospital managers in the Eastern Cape. The head of the Frere/CMH ethics committee has also received a copy of the research paper.

## Authors’ contribution

Authors contributed as follows to the conception or design of the work; the acquisition, analysis, or interpretation of the data for the work; and drafting the work or the revising it critically for important intellectual content: OB contributed 50 %/ LT contributed 20 %/DJVH contributed 20 %/MR contributed 10 %.

## Funding

The study was self funded with no company/external source of funding given.

## Declaration of competing interest

There was no conflict of interests declared by the authors.

## Appendix Supplementary materials

Image, application 1
