# Supplementary material for: The utilisation of emergency point-of-care ultrasound in a tertiary hospital emergency department in East London, South Africa
Source: Afr J Emerg Med. 2024 Jun 4;14(3):135–40. doi: 10.1016/j.afjem.2024.05.002 (PMC11214390; doi:10.1016/j.afjem.2024.05.002)
Supplement: Supplementary file 1 [file mmc1.docx]

Supplementary material:

Appendix: Audit forms

# eFAST

**ePoCUS audit form**

(*This form is used for documenting emergency informal U/S and **should not** replace formal radiological investigation)

Place patient sticker if available

1. Demographics

Date: Gender:

M

F

Time:

Date of birth: yyyy / mm / dd

1. ePOCUS provider credentialed?

Yes Provider signature:

No

1. Provisional diagnosis before performing ePoCUS

4. Provisional ED disposition before performing an ePoCUS application

d/c d/c & F/U OPD d/c & F/U CHC

specialist refer Emerg theatre Emerg review

5. ePOCUS application **Indication** **Findings**

eFAST Trauma (Blunt) Free fluid(abdo)

Trauma (penetrating) Pleural fluid

Medical Pericardial fluid

Obs/gyne Pneumothorax

Negative scan

Inconclusive scan

6. Additional ePOCUS findings/comments

7.Duration of study

<2 minutes 5-10 minute

2-5 minutes >10 minutes

8. Provisional diagnosis after performing ePoCUS

9. Provisional ED disposition after performing ePoCUS

d/c d/c & F/U OPD d/c & F/U CHC

specialist refer Emerg theatre Emerg review

**Thank you for completing the form 😊**

# B. ECHO

# ePoCUS audit form

(*This form is used for documenting emergency informal U/S and **should not** replace formal radiological investigation)

1. Demographics

Date:

Place patient sticker if available

Time:

M

F

Date of birth: yyyy / mm / dd Gender:

2. ePOCUS provider credentialed?

Yes Provider signature:

No

3. Provisional diagnosis before performing ePoCUS

4. Provisional ED disposition before performing an ePoCUS application

d/c d/c & F/U OPD d/c & F/U CHC

Specialist refer Emerg theatre Emerg r/v

5. ePOCUS application **Indication** **Finding**

ECHO SOB Pericardial effusion

Chest pains Tamponade

Abnormal ECG RV strain/dilation

CCF Reduced EF

Abn CXR(cardiomegaly) Clot

Inconclusive

Negative

6. Additional ePOCUS findings/comments

7.Duration of study

<2 minutes 5-10 minute

2-5 minutes >10 minutes

8. Provisional diagnosis after performing ePoCUS

9. Provisional ED disposition after performing ePoCUS

d/c d/c & F/U OPD d/c & F/U CHC

Specialist refer Emerg theatre Emerg r/v

**Thank you for completing the form 😊**

**C. Obs/Gyne**

**ePoCUS audit form**

(*This form is used for documenting emergency informal U/S and **should not** replace formal radiological investigation)

Place patient sticker if available

1. Demographics

Date:

M

F

Time: Gender:

Date of birth: yyyy / mm / dd

2. ePOCUS provider credentialed?

Yes Provider signature:

No

3.Provisional diagnosis before performing ePoCUS

4. Provisional ED disposition before performing an ePoCUS application

d/c d/c & F/U OPD d/c & F/U CHC

Specialist refer Emerg theatre Emerg r/v

5. ePOCUS application **Indication Findings**

Obs/Gyne Trauma(preg) Free fluid pelvis

Abdo pain(preg) Complete m/c

PVB (preg) Incomplete m/c

AUB(neg Preg) Ectopic

Pelvic mass Uterine/pelvic mass

Live fetus

Inconclusive

Negative

6. Additional ePOCUS findings/comments

7. Duration of study

<2 minutes 5-10 minute

2-5 minutes >10 minutes

8. Provisional diagnosis after performing ePoCUS

9.Provisional ED disposition after performing ePoCUS

d/c d/c & F/U OPD d/c & F/U CHC

Specialist refer Emerg theatre Emerg r/v

**Thank you for completing the form 😊**

# D. Hepatobiliary

# ePoCUS audit form

(*This form is used for documenting emergency informal U/S and **should not** replace formal radiological investigation)

1. Demographics

Date:

Place patient sticker if available

Time:

M

F

Date of birth: yyyy / mm / dd Gender:

2.ePOCUS provider credentialed?

Yes Provider signature:

No

3. Provisional diagnosis before performing ePoCUS

4. Provisional ED disposition before performing an ePoCUS application

d/c d/c & F/U OPD d/c & F/U CHC

Specialist refer Emerg theatre Emerg r/v

5. ePOCUS application **Indication** **Finding**

HPB Abdo pain Cholecystitis

Mass Gallstones

Pancreatic pathology Intra-abdominal mass

Jaundice Inconclusive

Negative

6. Additional ePOCUS findings/comments

7.Duration of study

<2 minutes 5-10 minute

2-5 minutes >10 minutes

8. Provisional diagnosis after performing ePoCUS

9. Provisional ED disposition after performing ePoCUS

d/c d/c & F/U OPD d/c & F/U CHC

Specialist refer Emerg theatre Emerg r/v

**Thank you for completing the form 😊**

# E. Lung

**ePoCUS audit form**

(*This form is used for documenting emergency informal U/S and **should not** replace formal radiological investigation)

Place patient sticker if available

1. Demographics

Date:

M

F

Time: Gender:

Date of birth: yyyy / mm / dd

2. ePOCUS provider credentialed?

Yes Provider signature:

No

3. Provisional diagnosis before performing ePoCUS

4. Provisional ED disposition before performing an ePoCUS application

d/c d/c & OPD F/U d/c & F/U CHC

Specialist refer Emerg theatre Emerg review

1. ePOCUS **Indication Findings**

Lung SOB Shred sign

Chest pain Pleural fluid

Abn CXR B-lines

Abn breath sounds Absent sliding/lung point

A-lines

Inconclusive

Negative

6.Additional ePOCUS findings/comments

7.Duration of study

<2 minutes 5-10 minute

- 1. minutes >10 minutes

8.Provisional diagnosis after performing ePoCUS

9.Provisional ED disposition after performing ePoCUS

d/c d/c & OPD F/U d/c & F/U CHC

Specialist refer Emerg theatre Emerg review

**Thank you for completing the form ☺**

# F. Procedure/Other(Ocular/MSK/Renal/Testes)

**ePoCUS audit form**

(*This form is used for documenting emergency informal U/S and **should not** replace formal radiological investigation)

1. Demographics

Place patient sticker if available

Date:

M

F

Time: Gender:

Date of birth: yyyy / mm / dd

2. ePOCUS provider credentialed?

Yes Provider signature:

No

3. Provisional diagnosis before performing ePoCUS

4. Provisional ED disposition before performing an ePoCUS application

d/c d/c & OPD F/U d/c & F/U CHC

Specialist refer Emerg theatre Emerg review

5. ePOCUS **Indication Findings**

Procedural CVC Successful

Thoracocentesis Unsuccessful

Pericardiocentesis

Peripheral IV

Other scans Ocular Findings:

Soft tissue/MSK

Testes

Renal

6.Additional ePOCUS findings/comments

7.Duration of study

<2 minutes 5-10 minute

- 1. minutes >10 minutes

8.Provisional diagnosis after performing ePoCUS

9.Provisional ED disposition after performing ePoCUS

d/c d/c & OPD F/U d/c & F/U CHC

Specialist refer Emerg theatre Emerg review

**Thank you for completing the form ☺**

# G. FASH

**ePoCUS audit form**

(*This form is used for documenting emergency informal U/S and **should not** replace formal radiological investigation)

1.Demographics

Place patient sticker if available

Date:

M

F

Time: Gender:

Date of birth: yyyy / mm / dd

2. ePOCUS provider credentialed?

Yes Provider signature:

No

3.Provisional diagnosis before performing ePoCUS

4. Provisional ED disposition before performing an ePoCUS application

d/c d/c & OPD F/U d/c & F/U CHC

Specialist refer Emerg theatre Emerg review

5. ePOCUS **Indication Findings**

FASH Constitutional sx in HIV Pleural effusion

HIV with respiratory sx Pericardial effusion

HIV with abdo sx Ascites

HIV with neurology sx Splenic microabscess

Suspected diss TB Para-aortic LN

Negative

Inconclusive

6.Additional ePOCUS findings/comments

7.Duration of study

<2 minutes 5-10 minute

- 1. minutes >10 minutes

8.Provisional diagnosis after performing ePoCUS

9.Provisional ED disposition after performing ePoCUS

d/c d/c & OPD F/U d/c & F/U CHC

Specialist refer Emerg theatre Emerg review

**Thank you for completing the form ☺**

# H. RUSH application

**ePoCUS audit form**

(*This form is used for documenting emergency informal U/S and **should not** replace formal radiological investigation)

1.Demographics

Place patient sticker if available

Date:

M

F

Time: Gender:

Date of birth: yyyy / mm / dd

2. ePOCUS provider credentialed?

Yes Provider signature:

No

3.Provisional diagnosis before performing ePoCUS

4. Provisional ED disposition before performing an ePoCUS application

d/c d/c & OPD F/U d/c & F/U CHC

Specialist refer Emerg theatre Emerg review

5. ePOCUS **Indication Findings**

RUSH protocol Undifferentiated shock Findings: **_______________**

Etiology:

Cardiogenic

Obstructive

Septic

Hypovolemic

Negative

Inconclusive

6.Additional ePOCUS findings/comments

7.Duration of study

<2 minutes 5-10 minute

- 1. minutes >10 minutes

8.Provisional diagnosis after performing ePoCUS

9.Provisional ED disposition after performing ePoCUS

d/c d/c & OPD F/U d/c & F/U CHC

Specialist refer Emerg theatre Emerg review

**Thank you for completing the form ☺**

1. **Table of MO breakdown**

| **Medical Officer experience at time of audit** | | | | | | |
| --- | --- | --- | --- | --- | --- | --- |
| MO | Work experience post community service year | ePoCUS experience | Scans | % | Scans with HOD removed | % |
|  |  |  |  |  |  |  |
| Credentialed |  |  |  |  |  |  |
|  |  |  |  |  |  |  |
| MO1 | 7 years | EMSSA Examiner | 156 | 31.7 | 0 | - |
| MO2 | 4 years | EMSSA Instructor/ Attended advanced course | 48 | 9.8 | 48 | 14.3 |
| MO3 | 3 years | EMSSA Instructor | 45 | 9.1 | 45 | 13.4 |
| MO4 | 2 years | EMSSA Instructor/Attended advanced course | 54 | 11.0 | 54 | 16.1 |
| MO5 | 2 years | EMSSA Credentialed | 32 | 6.5 | 32 | 9.5 |
| MO6* | 2 years | EMSSA Credentialed | 25 | 5.1 | 25 | 7.4 |
|  |  |  |  |  |  |  |
| Total |  |  | 360 | 73.2 | 204 | 60.7 |
|  |  |  |  |  |  |  |
| Non-credentialed |  |  |  |  |  |  |
|  |  |  |  |  |  |  |
| MO6* | 2 years | Attended core EMSSA ePoCUS | 16 | 3.3 | 16 | 4.8 |
| MO7 | 1 years | Attended core EMSSA ePoCUS | 65 | 13.2 | 65 | 19.3 |
| MO8 | 2 years | Attended core EMSSA ePoCUS | 21 | 4.3 | 21 | 6.3 |
| MO9 | 3 years | Attended core EMSSA ePoCUS | 20 | 4.1 | 20 | 6.0 |
| MO10 | 3 years | Attended core EMSSA ePoCUS | 10 | 2.0 | 10 | 3.0 |
| MO11 | 15 years | Attended core EMSSA ePoCUS | 0 | 0.0 | 0 | 0.0 |
| MO12 | 5 years | Attended core EMSSA ePoCUS | 0 | 0.0 | 0 | 0.0 |
|  |  |  |  |  |  |  |
| Total |  |  | 132 | 26.8 | 132 | 39.3 |
|  |  |  |  |  |  |  |
| Grand Total |  |  | 492 | 100.0 | 336 | 100.0 |

*- MO6 became credentialed during the audit and thus recorded findings as both a credentialed and non-credentialed provider.
